# Honokiol induces apoptosis of lung squamous cell carcinoma by targeting FGF2‐FGFR1 autocrine loop

**DOI:** 10.1002/cam4.1846

**Published:** 2018-12-05

**Authors:** Mengyuan Cen, Yinan Yao, Luyun Cui, Guangdie Yang, Guohua Lu, Liangjie Fang, Zhang Bao, Jianying Zhou

**Affiliations:** ^1^ Department of Respiratory Diseases, First Affiliated Hospital of Zhejiang University School of Medicine Zhejiang University Hangzhou China

**Keywords:** apoptosis, ERK, FGFR1, honokiol, lung SCC

## Abstract

Lung squamous cell carcinoma (SCC) accounts for a considerable proportion of lung cancer cases, but there is still a lack of effective therapies. FGFR1 amplification is generally considered a promising therapeutic target. Honokiol is a chemical compound that has been proven to be effective against various malignancies and whose analog has been reported to target the mitogen‐activated protein kinase family, members of a downstream signaling pathway of FGFR1. This was an explorative study to determine the mechanism of honokiol in lung SCC. We found that honokiol induced apoptosis and cell cycle arrest in lung SCC cell lines in a time‐ and dose‐dependent manner. Honokiol also restricted cell migration in lung SCC cell lines. Moreover, the expression of FGF2 and the activation of FGFR1 were both downregulated by honokiol. Pharmacological inhibition and siRNA knockdown of FGFR1 induced apoptosis in lung SCC cells. Our in vivo study indicated that honokiol could suppress the growth of xenograft tumors, and this effect was associated with the inhibition of the FGF2‐FGFR1 signaling pathway. In conclusion, honokiol induced cell apoptosis in lung SCC by targeting the FGF2‐FGFR1 autocrine loop.

## 1 INTRODUCTION

According to cancer statistics, lung cancer is one of the most frequently diagnosed cancers worldwide. Despite great improvement in treatments, lung cancer remains the leading cause of cancer mortality.^1^ Although in recent years there has been a decrease in the occurrence of lung squamous cell carcinoma (SCC), it still represents an estimated 30% of non‐small cell lung cancer (NSCLC) and is mainly attributed to tobacco consumption. As the majority of SCC tumors lack specific targetable mutations,^2^ the treatment for most advanced patients remains doublet chemotherapy containing platinum, which has limited effectiveness and apparent toxicity.

Fibroblast growth factor receptor1 (FGFR1) belongs to the receptor tyrosine kinase family, and its signaling pathway plays an important role in normal developmental and physiological processes such as proliferation, differentiation, survival, and the prevention of apoptosis.^3^ Dysregulation of the FGFR1 signaling pathway through gene amplification, chromosomal translocation, and point mutation has been described in various cancers.^4^, ^5^ FGFR1 amplification has been observed in 20% of pulmonary squamous carcinomas, which is a much higher frequency than that for other histological categories.^7^ In addition, this aberrant amplification is associated with poor prognosis and smoking.^8^, ^9^ Therefore, FGFR1 represents a promising target in lung SCC. Numerous clinical trials on FGFR1 inhibitors have been conducted recently. However, the problem of intrinsic and acquired resistance to monotherapy with these inhibitors has limited their use as therapeutic agents.^10^, ^11^ Therefore, alternative treatment options for lung SCC are urgently needed.

Honokiol, a promising bioactive compound purified from the traditional Japanese medicine magnolia, has been proven to be nontoxic and to have multifarious anticarcinogenic effects in a variety of cancer cell lines.^12^ Previous studies reported that honokiol‐mediated inhibition of cell proliferation and induction of cell apoptosis seemed to be associated with the suppression of nuclear factor‐κB (NF‐κB)^13^ and AKT,^14^ the downregulation of theBCL2 protein family^14^, ^15^ and the upregulation of proapoptotic proteins, such as Bax, Bak, Bad, and tBid.^14^, ^15^ However, a deeper understanding of the antitumor mechanism of honokiol should be sought. Magnolol, another compound derived from *Magnolia officinalis*whose chemical structure is similar to that of honokiol, had been identified for its antitumor function in lung SCC through the mitogen‐activated protein kinase (MAPK) family,^13^ which mediates signaling through FGFR1.^14^We hypothesize that honokiol inhibits proliferation and induces apoptosis in lung SCC cells by targeting FGFR1. To test our hypothesis, we assessed the chemotherapeutic effects of honokiol on lung SCC in vitro and in vivo.

## 2 MATERIALS AND METHODS

### 2.1 Cell lines and reagents

The human lung SCC cell SK‐MES‐1 was purchased from the Committee on Type Culture Collection of the Chinese Academy of Sciences (Shanghai, China). NCI‐H520 was obtained from Dr Ying (Department of Respiratory Diseases, Sir Run Run Shaw Hospital, Zhejiang University, Hangzhou, China). Both cell lines were maintained in RPMI‐1640 (Gibco BRL Co., Ltd., Houston, TX, USA) supplemented with 10% fetal bovine serum (FBS, Gibco BRL Co., Ltd.) at 37°C with 5% CO2. Honokiol was purchased from Sigma‐Aldrich (St. Louis, MO, USA), dissolved in DMSO at a concentration of 50 mmol/L and stored at −80°C. Soluble recombinant human FGF2 was purchased from PeproTech (Rocky Hill, NJ, USA). The FGFR1 inhibitor PD173074 was provided by Sigma‐Aldrich. Antibodies for various proteins were obtained from the following sources: cyclinD1, caspase‐3, cleaved caspase‐3,poly (ADP‐ribose) polymerase (PARP), cleaved PARP, phosphop44/42 MAPK (T202/Y204), 44/42 MAPK, and phosphopFGFR1 antibodies were purchased from Cell Signaling Technology (Danvers, MA, USA), while the FGFR1 antibody was purchased from Abcam (Cambridge, MA, USA).

### 2.2 Cell viability assay

Cells were seeded on a 96‐well plate at a density of 1 × 10^3^/well 24 hours before honokiol administration. Different doses of honokiol (0, 10, 20, 30, 40, 50, or 60 μmol/L) were added into each well, and the cells were incubated for 24, 48, 72, or 96 hours. Then, equal amounts of CCK8 (Dojindo Laboratories, Tokyo, Japan) were added into each well. After another two hours of incubation, the absorbance of the solutions was measured spectrophotometrically at 450 nm with an automatic microplate analyzer.

### 2.3 Cell cycle assay

Cells were starved for 24 hours before treatment with different dose of honokiol (0, 5, 10 and 20 μmol/L). After 96 hours, the cells were harvested, washed with PBS, fixed in 70% ethanol, and stored at −20°C for at least 24 hours. Once they were prepared for analysis, the cells were centrifuged at 500 *g* for 5 minutes, resuspended in 500 μL of PI/RNase staining buffer, incubated for 30 minutes at room temperature in the dark, and then analyzed using a BD FACSVerse (BD Biosciences, San Jose, CA, USA). The data were analyzed using FlowJo software Version 10.1.

### 2.4 Cell apoptosis assay

After drug administration, cells were harvested. For the detection of apoptosis, a FITC Annexin V Apoptosis Detection Kit and a PE Annexin V Apoptosis Detection Kit (BD Biosciences, Franklin Lakes, NJ, USA) were used according to the manufacturer's protocols. Briefly, the cells were washed twice with cold PBS and resuspended in binding buffer at a concentration of 1 × 10^6^ cells/mL before being stained with 5 µL of FITC Annexin V and 5 µL of propidium iodide or 5 µL of 7‐AAD and 5 µL of PE Annexin V. Then, the cells were incubated for 15 minutes at room temperature in the dark. Finally, apoptosis was analyzed with a BD FACSVerse (BD Biosciences, NJ, USA).

### 2.5 Cell migration assay

NCI‐H520 and SK‐MES‐1 cells (1 × 10^4^/200 µL) in FBS‐free RPMI‐1640 were seeded into the chambers (24‐well transwell chambers, 8‐µm pore size; Corning) with a complete culture medium, and culture medium with 20% FBS was added to the lower chamber as an attractant. After the NCI‐H520 and SK‐MES‐1 cells were incubated at 37°C in a 5% CO_2_environment for 24 and 48 hours, respectively, the cells that remained in the top chamber were removed with cotton swabs, and those that migrated to the underside of the filter were fixed with 4% paraformaldehyde and stained with 0.1% crystal violet. The number of cells was counted by bright field microscopy.

### 2.6 Immunoblotting

Cells and tissues were lysed with RIPA lysis buffer (Beyotime, Shanghai, China).A Pierce BCA Protein Assay Kit (Thermo Fisher Scientific, Rockford, IL, USA) was used to measure the protein concentration according to the manufacturer's instructions. Protein lysates were subjected to SDS‐PAGE and then transferred to polyvinylidene difluoride (PVDF) membranes (Millipore, Bedford, MA, USA). Enhanced chemiluminescence (ECL) was used to detect immunoreactive bands.^17^

### 2.7 Quantitative real‐time PCR

Total cellular RNA extraction was performed using a RNeasy Mini Kit (Qiagen, Hilden, Germany) according to the manufacturer's protocol, and RNA concentrations were measured with a NanoDrop 2000 (Thermo Scientific, Waltham, MA, USA). Synthesis of complementary DNA was performed by reverse transcription using a PrimeScript 1st Strand cDNA Synthesis Kit (Takara, Dalian, China) as recommended by the manufacturer. cDNA amplification was performed using a QuantiFast SYBR Green PCR Kit (Qiagen, Hilden, Germany), and gene expression was assessed with quantitative RT‐PCR^18^ Glyceraldehyde 3‐phosphate dehydrogenase (GAPDH) was used as an internal control to determine the relative expression of the target genes. The comparative Ct method (2^−ΔΔCt^) was used to analyze data. The specific primers for RT‐PCR are shown in Table 1.

**Table 1:** Primer sequences used for real‐time PCR

mRNA	Oligonucleotides (5′ to 3′)
FGF2‐F	5‐CGGCTGTACTGCAAAAACGG‐3
FGF2‐R	5‐GATGTGAGGGTCGCTCTTCTCC‐3
GAPDH‐F	5‐GGAGCGAGATCCCTCCAAAAT‐3
GAPDH‐R	5‐GGCTGTTGTCATACTTCTCATGG‐3

### 2.8 Transfection

After cultures reached 30%‐50% confluence, the medium was replaced with serum‐ and antibiotic‐free medium. Lung SCC cells were transfected with targeted siRNAs or negative control siRNA (GenePharma, Shanghai, China) using Lipofectamine 2000 (Invitrogen, Carlsbad, CA, USA) according to the manufacturer's instructions. At 48 hours after transfection, Western blot analysis was performed to determine the efficiency of inhibition. The siRNA sequences used for transfection are shown in Table 2.

**Table 2:** SiRNA sequences used for transfection

SiRNA	Oligonucleotides (5′ to 3′)
SiFGFR1‐1, sense	5‐CGGUCAUCGUCUACAAGAUdTdT‐3
SiFGFR1‐1, antisense	5‐AUCUUGUAGACGAUGACCGdTdT‐3
SiFGFR1‐2, sense	5‐GAUGGUCCCUUGUAUGUCAdTdT‐3
SiFGFR1‐2, antisense	5‐UGACAUACAAGGGACCAUCdTdT‐3
SiRNA‐NC, sense	5‐UUCUCCGAACGUGUCACGUdTdT‐3
SiRNA‐NC, antisense	5‐ACGUGACACGUUCGGAGAAdTdT‐3

### 2.9 Animal experiments

Three‐ to four‐week‐old female BALB/c‐nude mice (16‐18 g/mouse) were obtained from the Shanghai Experimental Animal Center (Chinese Academy of Sciences, Shanghai, China) and housed in the animal research center at the Traditional Chinese Medicine University of Zhejiang province. After one week of acclimatization, exponentially growing NCI‐H520 cells (5 × 10^5^ in 100 μL PBS/mouse) were injected subcutaneously in the right armpit of each mouse. Twenty‐four hours after tumor cell inoculation, animals were divided randomly into two groups with four mice per group. When palpable tumors arose, the mice in Group I were treated with 100 mg honokiol/kg body weight in 200 μL of 0.5% carboxymethylcellulose (w/v) and 0.025% Tween‐20 (v/v) in sterile water by oral gavage every three days. The dosage of honokiol was chosen based on two previous studies in which a significant inhibition of tumor xenograft growth was observed after honokiol administration.^15^, ^16^ In addition, the mice in Group II received the same volume of vehicle and served as a control group. The whole experiment was terminated 18 days after tumor cell injection, and during this period, mice were monitored for their body weight, food intake, and water consumption on a regular basis. Tumor size was measured with calipers through measurements of the two perpendicular diameters every three days using the formula: Volume = (width^2^ × length)/2.^19^ At the termination of the experiment, mice were sacrificed and the tumors were harvested. The volume of each tumor was measured according to the formula: Volume = (width^2^ × length)/2.^19^ All procedures were performed according to the Regulations for the Administration of Affairs Concerning Experimental Animals. The experiments were approved by the State Council of the People's Republic of China and the Experimental Animal Ethics Committee of Zhejiang University.

### 2.10 Statistical analysis

All data are presented as the mean ± SD of three independent experiments and were analyzed by SPSS 19.0 (SPSS Inc Chicago, IL, USA). The statistical significance of differences between the control and honokiol‐treated groups was calculated by Student's *t* test, and *P* < 0.05 was considered statistically significant.

## 3 RESULTS

### 3.1 Honokiol inhibits cell viability of lung SCC cells

**Figure 1:**
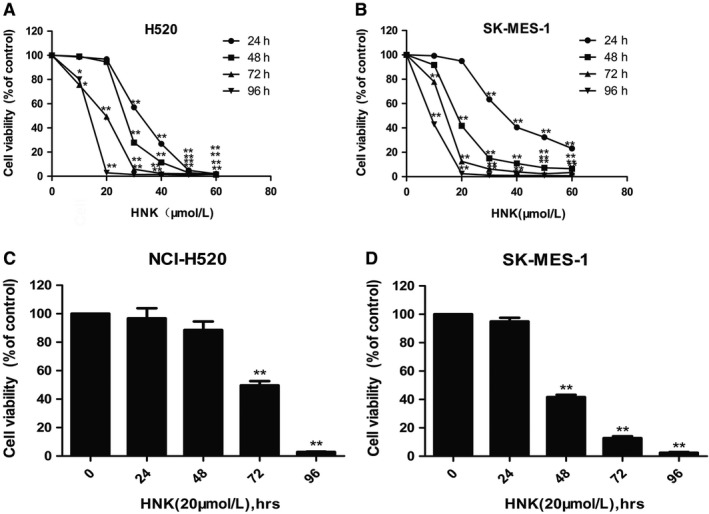
Honokiol inhibited the lung SCC cells proliferation in both dose‐dependent and time‐dependent manners. A and C, NCI‐H520 cells were incubated with 0‐60 µmol/L or 20 µmol/L honokiol for 24, 48, 72, and 96 hours. B, D, SK‐MES‐1 cells were incubated with 0‐60 µmol/L or 20 µmol/L honokiol for 24, 48, 72, and 96 hours. Cell viability was measured using CCK8 assays. Each experiment was performed in triplicate, thrice independently. The data are presented as mean ± SD. *P < 0.05, **P < 0.01 vs Control

After treatment with different concentrations of honokiol (0, 10, 20, 30, 40, 50, or 60 μmol/L) for 24, 48, 72, or 96 hours, both lung SCC cell lines showed significant reductions in cell viability in a time‐ and dose‐dependent manner after honokiol treatment, as shown in Figure 1. Increases in dose and treatment time decreased the viability of both H520 and SK‐MES‐1 cells, which suggested that honokiol is an effective against lung SCC. The 24, 48, 72, and 96 hours IC50 values (the concentration at 50% inhibition of cell viability) of honokiol were 32.21, 26.25, 17.27, and 12.20 μmol/L in H520 cells and 37.73, 18.54, 13.25, and 9.417 μmol/L in SK‐MES‐1 cells, respectively.

### 3.2 Honokiol induces cell proliferation and cell cycle arrest in lung SCC cells

**Figure 2:**
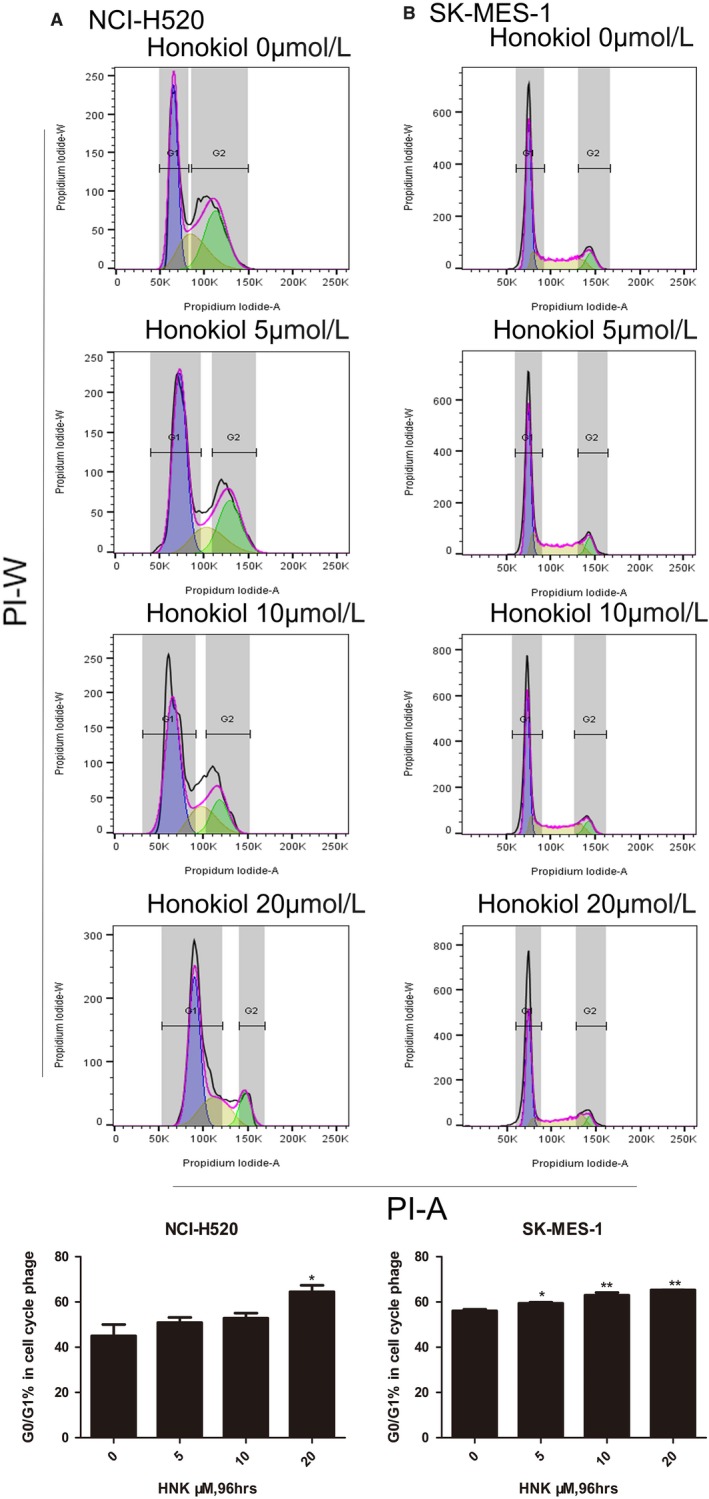
Honokiol induced G1 cell cycle arrest in human lung SCC cells. A, NCI‐H520, and (B) SK‐MES‐1 were treated with honokiol (0, 5, 10, and 20 µmol/L) for 96 hours. At the end of incubation, cells were collected for cell cycle distribution analyses by flow cytometry. Data were the mean ± SD. of triplicate samples. *P < 0.05, **P < 0.01 vs Control

To examine whether the inhibitory effect of honokiol led to growth arrest or cell death, the two cell lines were treated with a serial doses of honokiol for 96 hours, and flow cytometric analyses were performed.Our data revealed that low doses of honokiol induced significant dose‐dependent G1 growth arrest in H520 and SK‐MES‐1 cells Figure 2. Cyclin D1 has been implicated to play important roles in cell cycle regulation, affecting the proliferation of cells.^20^, ^21^ Cyclin‐dependent kinases (CDKs) form active complexes with D cyclins, including cyclins D1, D2, and D3, which further phosphorylate the retinoblastoma protein (RB) and drive the G1‐to‐S phase transition.^22^ The cyclin D/cyclin‐dependent kinases 4 and 6 (CDK4/6)‐RB pathway is believed to be of great importance in the proliferation of cancer cells.^23^ Therefore, the effect of honokiol on cyclin D1, CDK4, and RB was determined by Western blot analysis following the treatment of H520 and SK‐MES‐1 cells with different doses of honokiol for 96 hours. Western blot analysis revealed that treatment of these cells with honokiol (0, 7.5, 15, or 30 μmol/L) resulted in a concentration‐dependent decrease in the expression levels of cyclin D1 and CDK4 and the activation of RB (Figure 4).

### 3.3 Honokiol induces cell apoptosis in lung SCC cell lines

**Figure 3:**
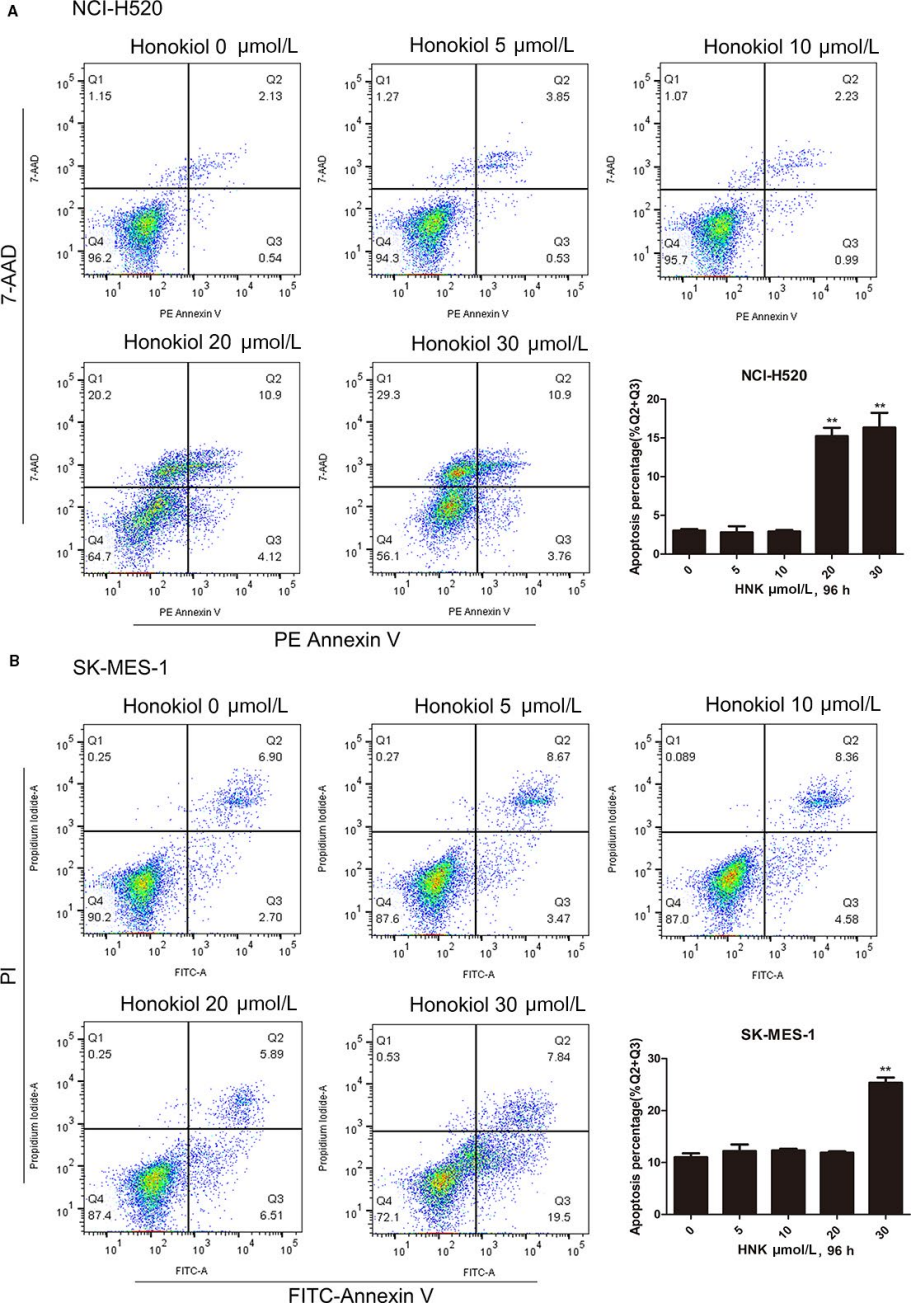
Honokiol induced the apoptosis of human lung SCC cells. A, NCI‐H520, and (B) SK‐MES‐1 were treated with honokiol (0, 5, 10, 20, and 30 µmol/L) for 96 hours. At the end of incubation, cells were collected and cell apoptosis was measured by flow cytometry. Data were the mean ± SD. of triplicate samples. *P < 0.05, **P < 0.01 vs Control

**Figure 4:**
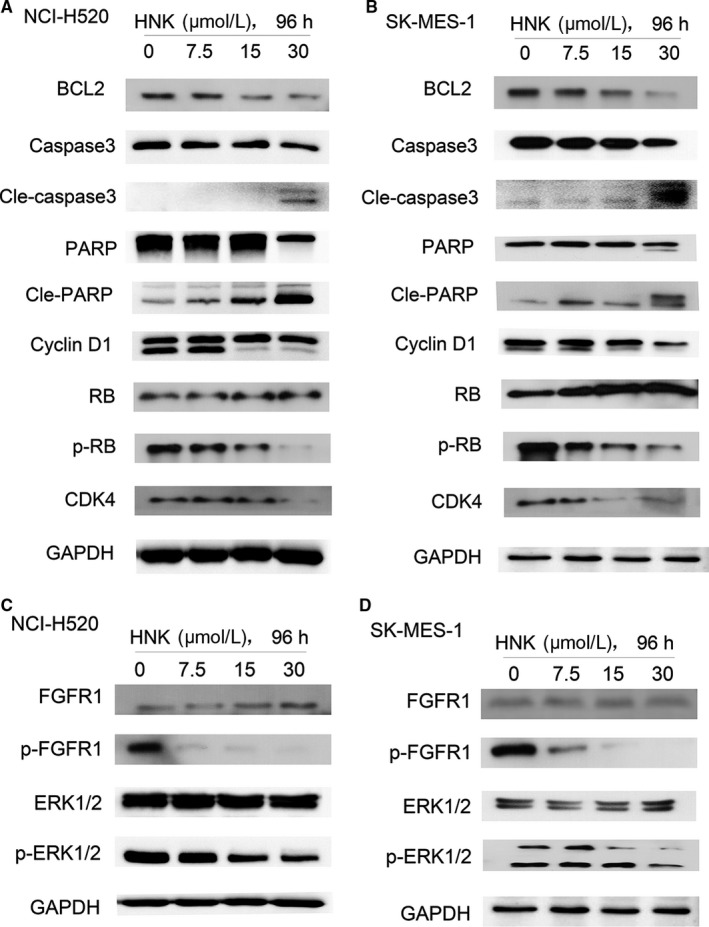
Honokiol exposure upregulated the expression of apoptosis factors, downregulated cell cycle promoting factors, and inhibited FGFR1 as well as its downstream signaling pathway. A and C, NCI‐H520 and (B, D) SK‐MES‐1 cells were incubated with different concentrations of honokiol for 96 hours, cell lysates were harvested, and the indicated proteins were determined by western blotting

Our data revealed that high doses of honokiol significantly and dose dependently induced apoptosis in lung SCC cell lines Figure 3. B cell lymphoma 2 (BCL2) and its family members (MCL1, BCLxL, BCLW, and BFL1) are important apoptotic regulators.^24^ Hence, we detected the expression of BCL2 to investigate the mechanism of cell apoptosis induced by honokiol. Furthermore, we did not detected any change in the expression of BAD (Figure S1). Caspase activation plays a central role in the execution of apoptosis. As the extrinsic and intrinsic apoptotic pathways merge at the caspase‐3 level, caspase‐3 is an ideal marker of apoptosis. H520 and SK‐MES‐1 cells had been incubated with honokiol for 96 hours before BCL2 and cleavage of caspase‐3 levels were determined by Western blot. BCL2 was inhibited in a dose‐dependent manner, while activation of caspase‐3 was observed at a high dose of honokiol (30 μmol/L), which was consistent with the outcome of flow cytometry. Furthermore, PARP, a downstream target of caspase‐3, was also activated Figure 4.

### 3.4 Honokiol suppressed FGFR1 and downstream targets in the FGFR1 signaling pathway in lung SCC cells

As it has been shown that amplification of FGFR1is observed in approximately 20% of lung SCCs,^7^ the effect of honokiol was determined on FGFR1 in lung SCC cells after treating the H520 and SK‐MES‐1 cell lines with honokiol for 96 hours. Western blot analysis indicated that treating H520 and SK‐MES‐1 with honokiol inhibited the phosphorylation of FGFR1.

Previous studies have indicated the RAS/(MEK/ERK) and PI3K/AKT/mTOR cascades as two main downstream signaling pathways of FGFRs.^25^ We investigated whether inhibition of FGFR by honokiol also affected the activation of ERK and AKT.As shown in Figure 4, phosphorylation of ERK was also inhibited in a dose‐related manner after incubation with honokiol. However, we did not detect any changes in AKT.

### 3.5 Honokiol restricted the migration of lung SCC cells

**Figure 5:**
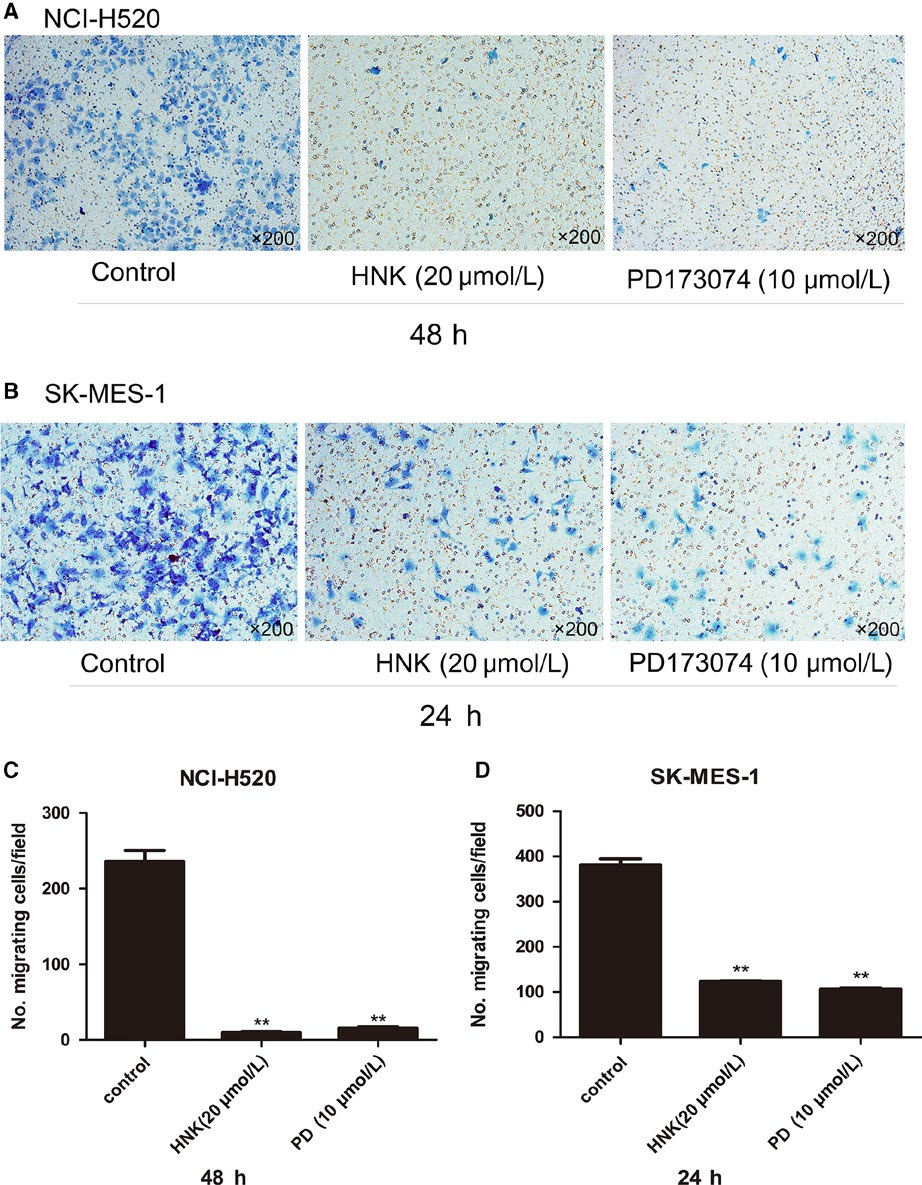
Honokiol inhibits migration of human lung SCC. After treating NCI‐H520 (A, C) and SK‐MES‐1 (B, D) with honokiol (20 µmol/L) or PD173074 (10 µmol/L) for either 48 hours or 24 hours, the cells that migrated to the underside of the filter were fixed and stained with 0.1% crystal violet, and counted in three randomly selected fields by bright field microscopy. Magnification, ×200. The data are representatives of three independent experiments and presented as mean ± SD. *P < 0.05, **P < 0.01 vs Control

Interestingly, we found that exposure to honokiol at a low concentration for 48 hours markedly suppressed the migration of H520 cells. Similar outcomes were also observed in SK‐MES‐1 cells after they were incubated with 20 μmol/L honokiol for 24 hours Figure 5. However, according to the CCK8 assay, the IC50 of honokiol was 26.25 μmol/L for H520 cells at 48 hours and 37.73 μmol/L for SK‐MES‐1 cells at 24 hours.

### 3.6 Honokiol downregulated the expression of FGF2 in lung SCC cells

**Figure 6:**
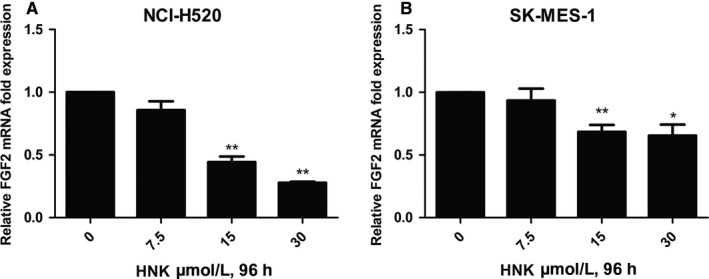
Honokiol inhibited the expression of FGF2 in human lung SCC cells. After (A) NCI‐H520 and (B) SK‐MES‐1 cells were treated with different doses of honokiol for 96 hours, the cells were harvested and total cellular RNA was extracted and analyzed using real‐time PCR. Data were the mean ± SD. of triplicate samples. *P < 0.05, **P < 0.01 vs Control

In addition to FGFR1 amplification, inappropriate expression of FGF ligands presents an alternative mechanism by which FGFRs can be activated and can participate in oncogenesis.^26^ Therefore, we measured the expression of FGF2 following treatment with honokiol in lung SCC cells. Quantitative real‐time PCR analysis presented a dose‐dependent decrease in FGF2 expression after honokiol treatment in both H520 and SK‐MES‐1 cells Figure 6.

### 3.7 Inhibition of FGFR1 increased apoptosis in lung SCC cells and downregulated activation of the ERK signaling pathway

**Figure 7:**
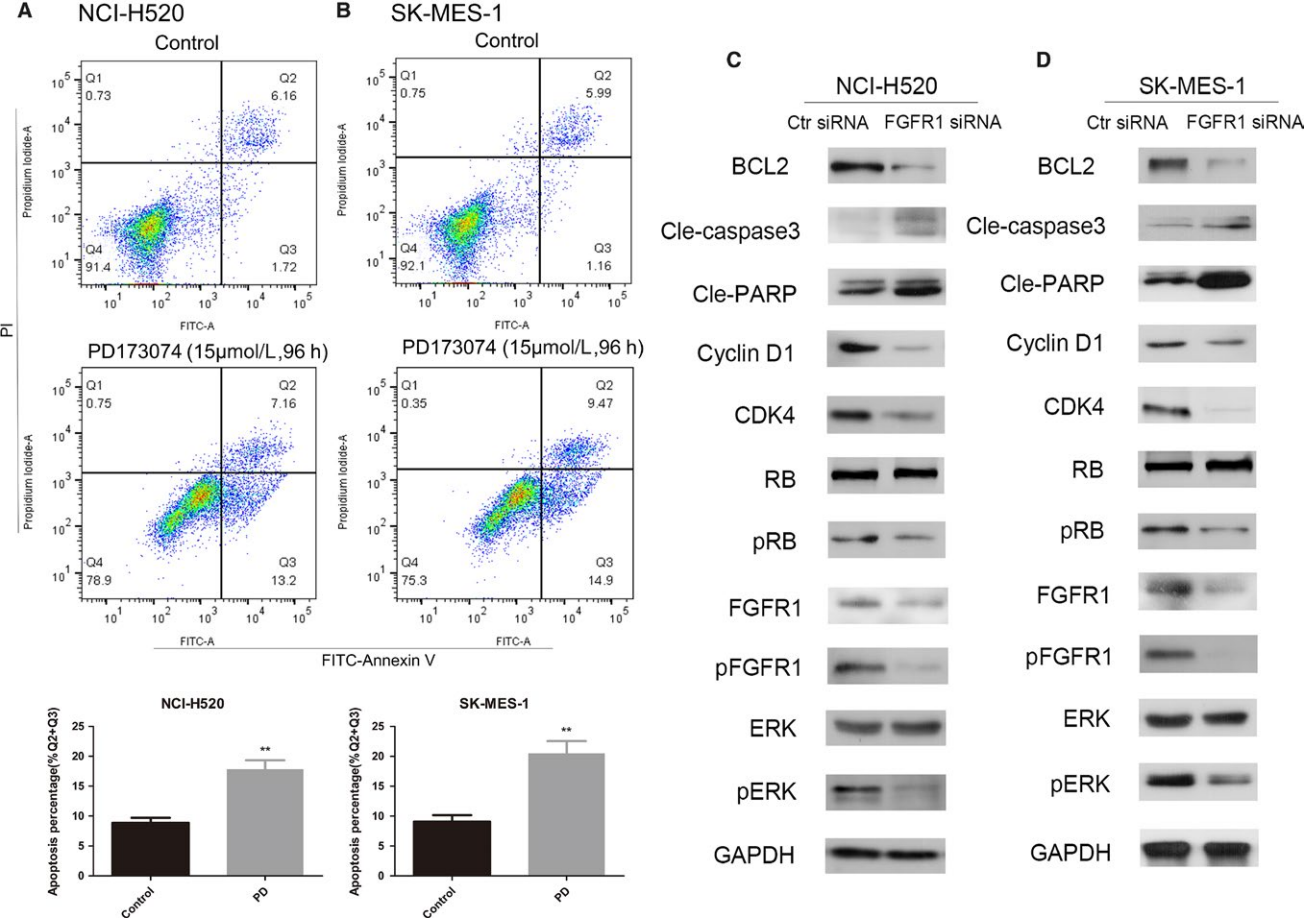
Honokiol suppressed FGFR1 and downstream targets in the FGFR1 signaling pathway in lung SCC cells. A, NCI‐H520 and (B) SK‐MES‐1 cells were incubated with PD173073 (15 µmol/L) for 96 hours before they were harvested and measured by flow cytometry for cell apoptosis. C NCI‐H520 and (D) SK‐MES‐1 cells were transfected with either FGFR1 siRNA or negative control siRNA for 48 hours before cells were harvested and the indicated proteins were determined by Western blotting. Data were the mean ± SD of triplicate samples. *P < 0.05, **P < 0.01 vs Control

To better understand the role of FGFR1 in honokiol‐mediated cell cycle arrest and apoptosis induction, we conducted both pharmacological inhibition and siRNA knockdown of FGFR1 in lung SCC cell lines. PD173074 is an ATP pocket inhibitor that shows both high affinity and selectivity for FGFR1.^27^ To investigate the role of the FGFR1 signaling pathway in the apoptosis‐induction effect of honokiol on H520 and SK‐MES‐1 cells, we detected apoptosis in both cell lines after PD173074 treatment. The proportion of apoptotic cells was significantly increased in the PD173074‐treated groups compared to the control group, as shown in Figure 7A,B. Knockdown of FGFR1 using siRNA significantly inhibited the phosphorylation of ERK. In addition, the cell cycle‐related proteins cyclin D1, CDK4, and RB were inhibited by siRNA knockdown of FGFR1, while apoptosis‐related proteins such as caspase‐3 and PARP were activated Figure 7C,D).

### 3.8 FGF2 attenuated honokiol‐mediated apoptosis induction

**Figure 8:**
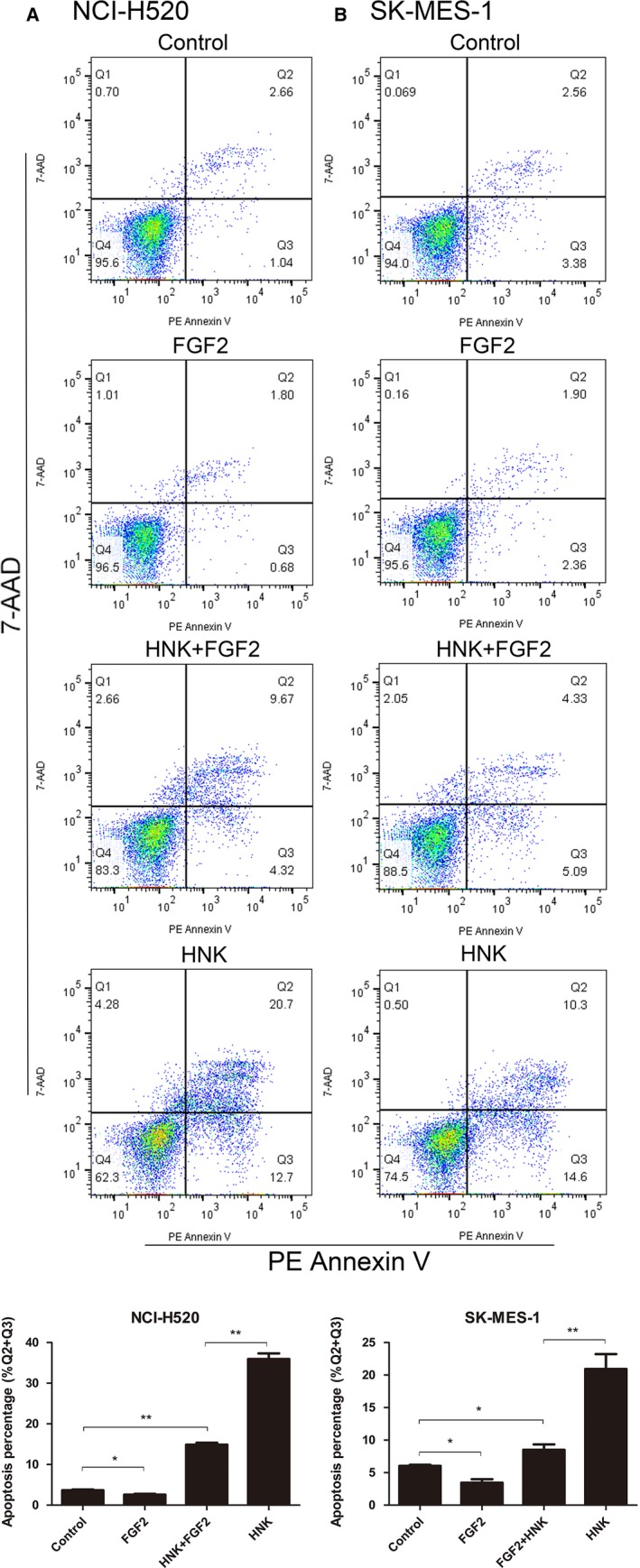
FGF2 reduced the apoptosis of human lung SCC cells and inhibited the apoptosis‐inducing function of honokiol. A NCI‐H520 and (B) SK‐MES‐1 cells were treated with DMSO control, FGF2 (10 ng/mL), FGF2 (10 ng/mL) in combination with honokiol (30 µmol/L) ，which was added 1 hour before, and honokiol (30 µmol/L), respectively, for 96 hours and then they were collected and measured by flow cytometry for cell apoptosis. Data were the mean ± SD. of triplicate samples. *P < 0.05, **P < 0.01 vs Control

To confirm whether honokiol inhibited FGFR1 activation through downregulation of FGF2 or by directly binding to the receptor, we detected apoptosis in H520 and SK‐MES‐1 cells after they were incubated with DMSO control; FGF2 (10 ng/mL) only; FGF2 (10 ng/mL) in combination with honokiol (30 μmol/L), which was added an hour earlier; or honokiol (30 μmol/L) only. FGF2 administration significantly reduced apoptosis in both H520 and S‐MES‐1 cells. Compared to that in the cells receiving the honokiol‐only treatment, the apoptosis rate in the cells treated withFGF2 and honokiol together was much lower Figure 8. These results may suggest that FGF2 reduced cell apoptosis and attenuated honokiol‐mediated apoptosis induction in lung SCC cell lines.

### 3.9 Honokiol exerted antitumor effects in the NCI‐H520 xenograft nude mouse model

**Figure 9:**
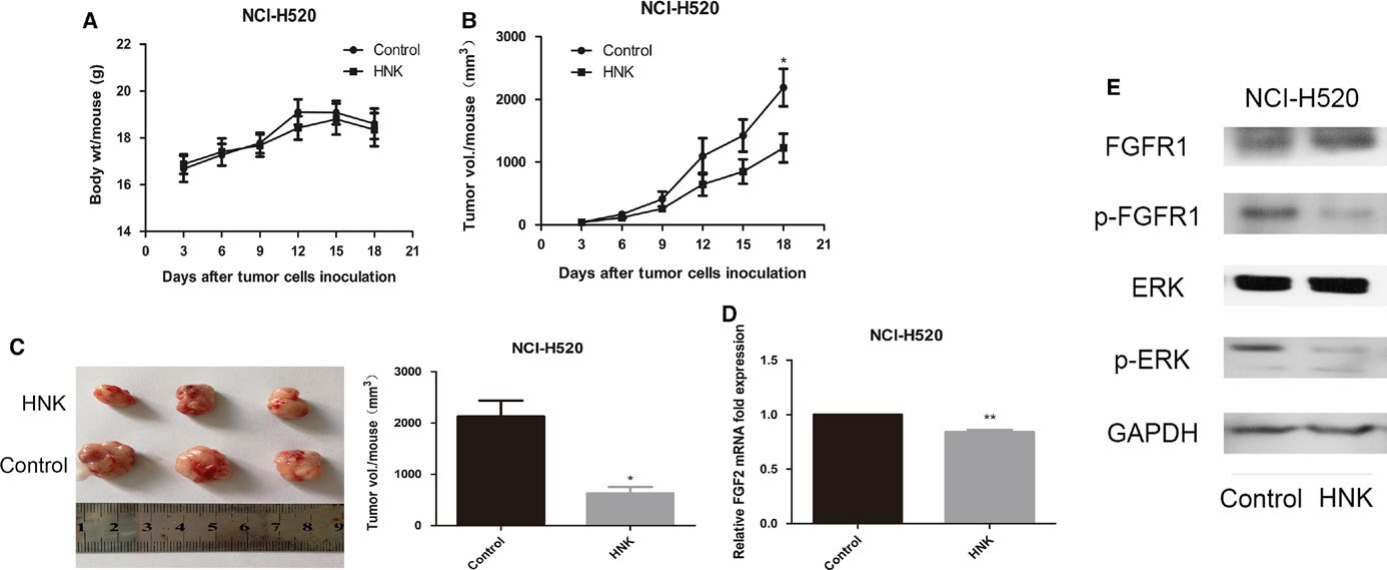
In vivo antitumor effects of honokiol in NCI‐H520 xenograft models. Three‐ to four‐week‐old female BALB/c‐nude mice were injected subcutaneously with equal numbers of NCI‐H520 cells and then divided randomly into two groups with four mice per group. When palpable tumors arose, mice in Group I were treated with 100 mg honokiol/kg body weight of mouse in 200 µL of 0.5% carboxymethylcellulose (w/v) and 0.025% Tween‐20 (v/v) solved in sterile water by oral gavage and mice in Group II received the same volume of vehicle every three days. The whole experiment was terminated 18 days after tumor cells injection. A and B, Body weight of mice and tumor volume were determined every three days after the onset of treatment. C, On day 18, the tumors were carefully dissected from the mice and the volumes of tumors were measured. (D) Expression of FGF2 was detected in the harvested tumors by real‐time PCR. E, The activation of FGFR1 signaling pathway in tumors was determined by Western blotting

To determine whether the antitumor effect of honokiol observed in vitro was preserved in vivo, we constructed NCI‐H520 xenograft models. The mice that were administered honokiol did not exhibit impaired movement or any other visible signs of physical toxicity. The average body weight of the honokiol‐treated and the nonhonokiol‐treated control mice remained almost identical throughout the 18‐day duration of the experimental Figure 9A).

According to the measurements of tumor size, the treatment of mice with honokiol resulted in reduced growth of H520 xenografts compared with that in the vehicle‐treated control mice Figure 9B). At the termination of the experiment on the 18th day, the mice were sacrificed, their tumors were harvested, and the tumor volume per mouse in each treatment group was calculated according to the tumor's length and width. The volume of the H520 tumors was lower in mice administered honokiol than in nonhonokiol‐treated control mice Figure 9C).

### 3.10 Honokiol downregulated the expression of FGF2 and inhibited the activation of the FGFR1 and ERK signaling pathways in lung SCC tumor xenografts

To evaluate whether the inhibition of lung SCC tumor xenograft growth by honokiol was associated with the downregulation of FGF2, we determined the levels of FGF2 in the mRNA extracted from tumor samples by RT‐PCR. Treatment of mice with honokiol inhibited the levels of FGF2 in tumor xenograft samples compared to those in the tumor samples from the control mice Figure 9D). Furthermore, Western blot analysis indicated that honokiol treatment also decreased the levels of pFGFR1and pERK in tumors, while the levels of FGFR1and ERK remained unchanged Figure 9E). These results suggested the involvement of these molecular targets in the honokiol‐mediated prevention of the in vivo growth of lung SCC xenografts in mice.

## 4 DISCUSSION

To our knowledge, there have been few studies regarding the antineoplastic activity of honokiol in lung SCC. The present study indicated that honokiol could significantly and dose‐dependently induce G0/G1 cell cycle arrest and apoptosis as well as inhibit migration in lung SCC cell lines. Furthermore, we determined the expression of FGF2 and the activation of FGFR1, which were both downregulated by honokiol. Pharmacological inhibition of FGFR1 with PD173074 induced apoptosis in both SCC cell lines. SiRNA knockdown of FGFR1 downregulated the ERK signaling pathway and cell cycle‐related proteins and activated cell apoptosis. The in vivo study indicated that honokiol could inhibit the growth of xenograft tumors, and this effect was associated with the inhibition of the FGF2‐FGFR1 signaling pathway.

Dysregulation of cell proliferation plays an important part in neoplastic growth. Our in vitro data showed that honokiol affected cell cycle regulatory proteins. Cyclin D1, together with other cyclins, plays an important role in cell cycle control. Previous studies have reported that cyclin D1 can regulate the G1‐to‐S phase transition.^28^ Overexpression and/or amplification of cyclin D1 has been described in various kinds of human tumors, including pancreatic cancer, non‐small cell lung carcinoma, and breast cancer.^29^, ^30^ CDKs are believed to be partner kinases of the D‐type cyclins that form holoenzymes with the D‐type cyclins and then monophosphorylate the RB protein, hence inducing progression through the G1 phase of the cell cycle.^23^ Treating lung SCC cells with honokiol markedly reduced the expression of cyclin D1 and CDK4 and therefore regulated the proliferation of lung SCC cell lines. BCL2 is an antiapoptotic protein in the BCL2 family that inhibits the activation of proapoptotic BCL2 family proteins in the absence of apoptotic stimuli. Thus, overexpression of BCL2 prevents cells from undergoing apoptosis, which contributes to the genesis and progressing of malignancy.^34^ Caspase‐3 has been recognized as one of the effector caspases in cell apoptosis.^35^ Activation of caspase‐3 and its downstream factors by honokiol indicated an apoptosis‐regulating function of honokiol in lung SCC, which is considered as a protective mechanism against cancer progression.^36^ Our study indicated that honokiol administration could downregulate BCL2 expression, promote the caspase‐3 signaling pathway, and accelerate cell apoptosis. Cell migration is an important process in tumor metastasis and progression.^37^ Several studies have reported that honokiol can inhibit the migration of various kinds of malignancies under different mechanisms.^37^, ^38^, ^39^ To investigate whether honokiol had a similar function in lung SCC, we performed a cell migration assay and found that honokiol could significantly inhibit the migration of H520 and SK‐MES‐1 cells, as could PD173074, which might suggest that inhibition of FGFR1 can restrict the migration of lung SCC.

The signaling of FGFs through FGF receptors (FGFRs) has been implicated as an autocrine signaling loop that leads to tumor proliferation and angiogenesis in a variety of NSCLC cell lines and is potentially a mechanism of resistance to both anti‐VEGF and anti‐EGFR therapies.^40^ Previous studies have revealed that overexpression of FGF2is associated with poor prognosis in NSCLC patients.^41^, ^42^ In our preliminary experiment, lung SCC cell lines were more sensitive to honokiol two adenocarcinoma cell lines, A549 and H1299, used in another study.^15^ As previous studies have revealed that overactivation of the FGF2‐FGFR1 autocrine loop occurs mainly in lung SCC, we hypothesized that the antitumor function of honokiol might be related to the inhibition of the FGF2‐FGFR1 pathway.^25^ Our study demonstrated that treatment of SCC cell lines with honokiol decreased the expression of FGF2 and inhibited the activation of FGFR1 and its downstream molecule ERK. To confirm the role of the FGF2‐FGFR1 autocrine loop in the antitumor function of honokiol, we used PD173074, a selective FGFR1 inhibitor, and found that it induced significant apoptosis in SCC. Using siRNA‐mediated knockdown of FGFR1, we further investigated the activation of the related signaling pathway and discovered dephosphorylation of ERK, inhibition of the cyclin D1/CDK4/RB pathway and activation of the cell apoptosis pathway, which suggested that inhibition of FGFR1 might be one of the mechanisms underlying the antineoplastic function of honokiol.

To further investigate how honokiol inhibited FGFR1, we incubated NCI‐H520 and SK‐MES‐1 cells with both FGF2 and honokiol and found that the apoptosis rate of the dual‐treated cells was markedly reduced compared to that of the honokiol‐treated group. These results suggested that honokiol might suppress the FGF2‐FGFR1 autocrine loop signaling pathway by inhibiting the expression of FGF2.It has been reported that NF‐κB may serve as a transcription factor for the FGF2 gene, regulating the expression and release of FGF2.^44^, ^45^ In our investigation, we found considerable evidence that honokiol could inhibit the activation of the NF‐κB pathway.^46^, ^47^ Hence, we infer that honokiol reduces the expression of FGF2 via the NF‐κB signaling pathway. However, the fact that the treatment of the lung SCC cell lines with both honokiol and FGF2 still induces apoptosis implies that inhibition of FGF2 is not the only mechanism underlying the antitumor function of honokiol.^49^, ^50^, ^51^

The therapeutic effect of honokiol against lung SCC was further examined and verified using an in vivo model with tumor xenografts. The outcome of this study provided evidence that administration of honokiol by oral gavage inhibited the growth of NCI‐H520 tumor xenografts without any apparent signs of toxicity in mice. This result is consistent with the results of two other studies that used NSCLC or HNSCC cell lines as tumor xenograft models.^15^, ^16^ In further investigation, honokiol inhibited FGF2 expression and activation of the FGFR1 signaling pathway in tumor xenografts. This suggests that the inhibitory effect of honokiol on tumor xenograft growth may be related to the downregulation of FGF2 and the inhibition of FGFR1.

In conclusion, honokiol dose‐dependently induced G0/G1 cell cycle arrest and cell apoptosis in lung SCC cells, and the apoptosis‐inducing function of honokiol seems to be related to the inhibition of the FGF2‐FGFR1 pathway.
