# Supplementary figures and images for: Honokiol induces apoptosis of lung squamous cell carcinoma by targeting FGF2‐FGFR1 autocrine loop
Source: Cancer Med. 2018 Dec 5;7(12):6205–18. doi: 10.1002/cam4.1846 (PMC6308115; doi:10.1002/cam4.1846)

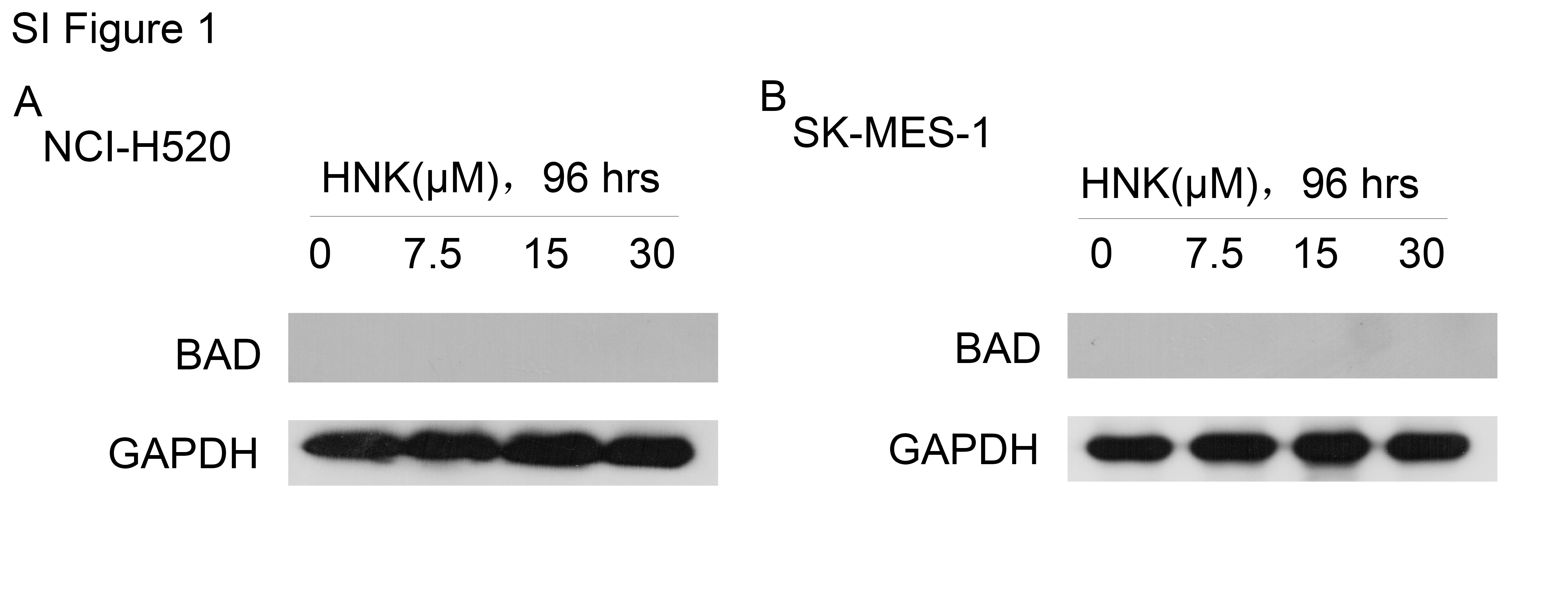

Supplement: Supplementary file 1 [file CAM4-7-6205-s001.tif]
